# Preventing musculoskeletal injuries among recreational adult volleyball players: design of a randomised prospective controlled trial

**DOI:** 10.1186/s12891-017-1699-6

**Published:** 2017-08-02

**Authors:** Vincent Gouttebarge, Johannes Zwerver, Evert Verhagen

**Affiliations:** 1 Dutch Consumer Safety Institute, 1062 XD, Amsterdam, The Netherlands; 2 Academic Center for Evidence based Sports medicine (ACES), Academic Medical Center, Amsterdam, The Netherlands; 3 UCT/MRC Research Unit for Exercise Science and Sports Medicine (ESSM), Department of Human Biology, Faculty of Health Sciences, University of Cape Town, Cape Town, South Africa; 4 Amsterdam Collaboration for Health & Safety in Sports, Academic Medical Center & VU University Medical Center, Amsterdam, The Netherlands; 5 Center for Sports Medicine, University of Groningen, University Medical Center Groningen, Groningen, The Netherlands; 6 Department of Public and Occupational Health, VU University Medical Center, Amsterdam Movement Sciences, Amsterdam, The Netherlands; 7 Australian Centre for Research into Injury in Sport and its Prevention, Federation University Australia, Ballarat, VIC Australia

**Keywords:** Volleyball, Musculoskeletal injuries, Prevention, Intervention mapping

## Abstract

**Background:**

Both acute and overuse injuries are common among recreational volleyball players, especially finger/wrist, ankle, shoulder and knee injuries. Consequently, an intervention (‘VolleyVeilig’) was developed to prevent or reduce the occurrence of finger/wrist, shoulder, knee and ankle injuries among recreational volleyball players. This article describes the design of a study evaluating the effectiveness of the developed intervention on the one-season occurrence of finger/wrist, shoulder, knee and ankle injuries among recreational adult volleyball players.

**Methods:**

A randomized prospective controlled trial with a follow-up period of one volleyball season will be conducted. Participants will be healthy recreational adult volleyball players (18 years of age or older) practicing volleyball (training and/or match) at least twice a week. The intervention (‘VolleyVeilig’) consists of a warm-up program based on more than 50 distinct exercises (with different variations and levels). The effect of the intervention programme on the occurrence of injuries will be compared to volleyball as usual. Outcome measures will be incidence of acute injury (expressed as number of injuries per 1000 h of play) and prevalence of overuse injuries (expressed as percentage).

**Discussion:**

This study will be one of the first randomized prospective controlled trials evaluating the effectiveness of an intervention on the occurrence of both acute and overuse injuries among recreational adult volleyball players. Outcome of this study could possibly lead to the nationwide implementation of the intervention in all volleyball clubs in The Netherlands, ultimately resulting in less injuries.

**Trial registration:**

Dutch Trial Registration NTR6202, registered February 1st 2017. Protocol: Version 3, February 2017.

## Background

Volleyball is one of the most popular sport in the world [1]. Besides its beneficial health effects, volleyball is also associated with a risk for musculoskeletal injuries, either acute or overuse injuries [2–4]. The incidence of musculoskeletal injuries among volleyball players ranges from 1.7 to 10.7 injuries per 1000 playing hours, occurring mostly in the fingers/wrists, shoulders, knees and ankles [2–4]. These injuries might induce impairments in daily life, sport and/or work and lead to substantial direct and indirect healthcare costs [5]. Consequently, integral measures to prevent or reduce the number of musculoskeletal injuries among volleyball players are needed.

Accordingly, a scientific research project together with the Dutch Volleyball Federation (Nevobo) has started in The Netherlands (funded by ZonMW, the Netherlands Organization for Health Research and Development). Aims of this project are (i) to develop an evidence-based intervention to prevent the occurrence of finger/wrist, shoulder, knee and ankle injuries among young and adult recreational volleyball players, and (ii) to evaluate the effectiveness of the developed intervention. After the systematic development of ‘VolleyVeilig’ as an intervention to prevent the occurrence of musculoskeletal injuries among recreational volleyball players, the next step is to assess its effectiveness within a randomised prospective controlled trial. This article describes the design of such a study.

## Methods/design

The CONSORT statement is followed to describe the design of the study [6]. This statement is a checklist intended to improve the quality of reports of randomized controlled trials.

### Objective and hypothesis

The objective of the study is to evaluate the effectiveness of the developed intervention (‘VolleyVeilig’) on the one-season occurrence of finger/wrist, shoulder, knee and ankle injuries among recreational adult volleyball players. Analogous to previous studies about the effect of preventive interventions in volleyball [7] and in other sports [8, 9], the hypothesis of this study is that the developed intervention will lead to a 40% reduction of the number of musculoskeletal injuries over the one-season follow-up in the intervention group by comparison to the control group.

### Study design and allocation

**Fig 1:**
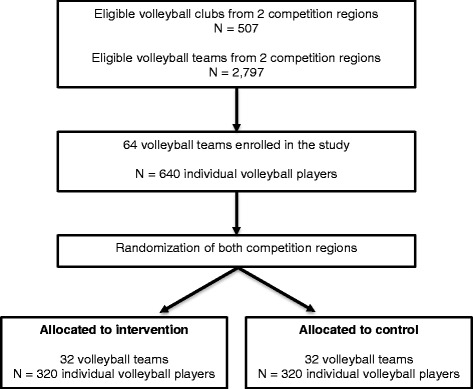
Anticipated flowchart of participants in the randomized prospective controlled trial

A randomized prospective controlled trial with a follow-up period of one volleyball season (September, 2017 – June, 2018) will be conducted (Fig. 1). In The Netherlands, Nevobo organizes its recreational competitions in 4 different geographical regions and recreational teams compete against each other within a geographical region. For practical reasons, the two largest competition regions (geographical regions West and East) will be selected for the study and randomly assigned to an intervention region and a control region. This is done to avoid any contamination and competitive bias within a competition region. The expectation is that coaches might be more inclined to participate if the group allocation and thus the volleyball conditions (training and match) do not differ between teams within a competition region. The need for ethical approval was waived by the Medical Ethics Review Committee of the Academic Medical Center because the Medical Research Involving Human Subjects Act (WMO) does not apply to our study protocol (W17_048#17.065; Amsterdam, The Netherlands). The study protocol is registered in the Dutch Trial Registry (ID: NTR6202).

### Participants and recruitment

The participants will be healthy recreational adult volleyball players. Inclusion criteria are: (i) 18 years of age or older; (ii) playing in a volleyball team competing recreationally in a competition region involved in the study; (iii) practicing volleyball (training and/or match) at least twice a week; (iv) speaking and reading Dutch fluently; (v) owning an email address.

Between March and May 2017, coaches of eligible teams will be contacted by the Nevobo and will receive detailed information about the study (purpose and procedures). Subsequently (August 2017), coaches potentially interested in the study will be informed face to face during explanatory group meetings about the purpose of the study, the procedures of the study and their study group allocation. At these explanatory meetings, coaches receive invitation letters about the purpose and procedures of the study for their players. Coaches and players willing to be enrolled in the study will give their informed consent prior the start of the 2017–2018 season, agreeing then to participate voluntarily in the study. The flow of the participants is presented in Fig. 1.

### Sample size

Analogous to previous studies about the effect of preventive interventions in volleyball [7] and in other sports [8, 9], the assumption is that a 40% injury reduction will be achieved over one season in the intervention group by comparison to the control group. To achieve 80% power with a significance level of 0.05, an injury prevalence estimate of 0.25 in the control group and a loss to follow-up among players of 15% over one season (no multiple cluster randomization), the sample size calculation reveals that 640 volleyball players are needed in the study [4]. Consequently, we will strive to enroll 64 teams (average of 10 players per team) assigned either to the intervention or the control group (equal number of 32 teams in each group). The teams within the intervention and control groups will be matched according to their competition level.

### Intervention

**Fig. 2:**
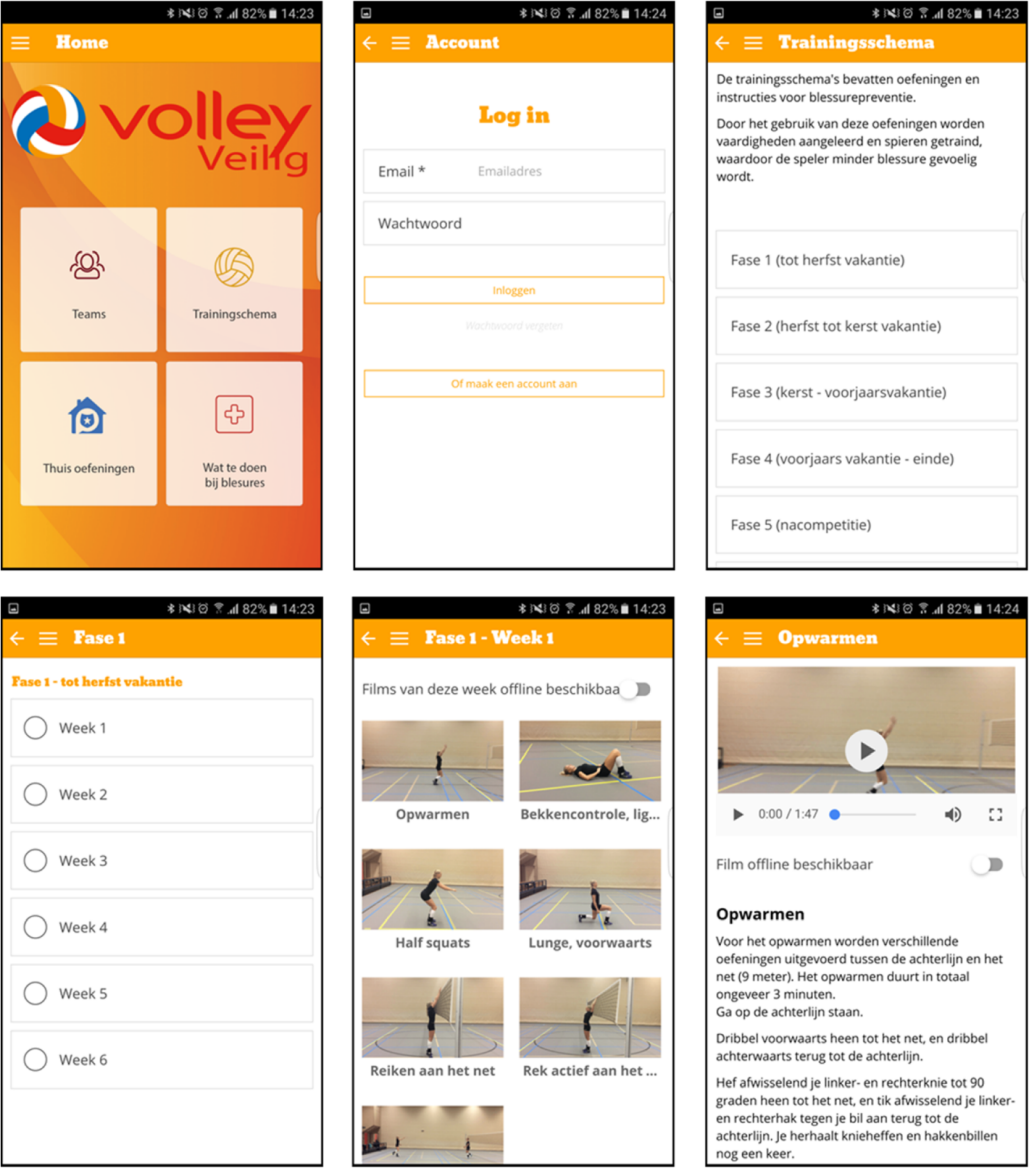
The intervention for volleyball players

The intervention ‘VolleyVeilig’ (in Dutch) is made available exclusively to the participants within the intervention group, aiming to prevent or reduce the occurrence of finger/wrist, shoulder, knee and ankle injuries among adult recreational volleyball players. The intervention consists of a warm-up program including more than 50 distinct exercises (with different variations and levels) that should be conducted at least twice a week prior to any volleyball activity (training or match) [10]. The intervention is divided into 6 phases, each lasting 5 to 6 weeks (accordingly to a typical volleyball season), providing volleyball coaches every week with a new warm-up program that shows progressive increments in terms of intensity, frequency, duration and/or complexity. Each warm-up program lasts 15 min and is divided into a preparatory cardiovascular warm-up (2 to 3 min), core stability exercises (2 to 3 min; for instance straight plank), exercises principally directed towards the prevention of knee injuries (4 to 5 min; for instance squat), and exercises principally directed towards the prevention of shoulder injuries (4 to 5 min; for instance external rotation strength with resistance elastic). The exercises directed towards the prevention of ankles injuries (for instance one legged stance) are embedded within each warm-up program. All warm-up programs of the intervention are overseen by the coaches, being available through a website and an application for smartphone/tablet (automatic synchronisation) that are accessible with a username and password. Information and instructions about the exercises are available as texts and video’s (including voice-over). Illustrations of the website and application for smartphone/tablet as well as examples of exercises within the intervention are presented in Fig. 2.

### Volleyball as usual

The teams and players in the control group will not have access to the intervention and will be asked to perform their warming up and volleyball activities as usual.

### Exposure and injury monitoring

Coaches will be asked every two weeks during the 2017–2018 season to retrospectively report the details of their players’ participation for each training and match. Therefore, an online form sent by email will be used (anonymous and coded for privacy reasons). A reminder will be sent to coaches after approximately four days in case of no response. Once completed, the online forms will be saved automatically on an electronic server secured according to good clinical practice with forced strong passwords, Secure Sockets Layer security and regular backups.

Players will be asked every two weeks during the 2017–2018 season to complete the translated and modified Dutch version of the Oslo Sports Trauma Research Centre questionnaire (OSTRC) [11, 12]. The OSTRC questionnaire has been proposed and validated to register and monitor sports-related health problems over time, including acute and overuse injuries [13]. The internal consistency (Cronbach’s a) of the OSTRC questionnaire is estimated at 0.96 and 0.91 for overall problems (including illnesses) and overuse injuries, respectively [11, 13]. With this questionnaire, both acute and overuse injuries are registered through four key questions related to: (i) difficulties to participate normally in volleyball activity, (ii) reduced training volume due to injury or complaint, (iii) influence of injury or complaint on performances, and (iv) experienced complaint i.e. symptoms during volleyball during the previous 2 weeks. If no problems are reported on these four key questions, the questionnaire is finished. If an injury or complaint is reported, subsequent questions are asked about location, circumstances, recurrence and return to play. In the present study, acute injury is defined as a sudden event sustained by a player during a volleyball activity (training or match) that results in the player to stop his or her volleyball activity [14, 15]. Overuse injury is defined as a non-sudden event gradual in onset sustained by a player due to participation in volleyball that results in reduced performance and/or ultimately in the player to decrease or stop his or her volleyball activity (training or match) [14, 15]. For the registration of injury, an online form sent by email will be used (anonymous and coded for privacy reason). A reminder will be sent to players after approximately four days in case of no response. Once completed, the online forms will be saved automatically on an electronic server secured according to good clinical practice with forced strong passwords, SSL security and regular backups.

### Adherence

Being an important construct in effectiveness studies and referring to the degree that teams and athletes follow the prescribed intervention, adherence to VolleyVeilig will be monitored during the 2017–2018 season [16]. Therefore, the coaches allocated to the intervention group will be asked to report every two weeks for each session (training and match) and for each player whether VolleyVeilig is used and the number of VolleyVeilig exercises performed per session. These questions will be embedded in the online form used to monitor the players’ exposure. In addition to the coaches’ report, study team members will make random unannounced visits to the teams allocated to the intervention group and will observe the execution (or not) of VolleyVeilig at the beginning of a session (training or match) from a location where the coaches are unaware of this observation. To explore the warm-up activities performed in the control group, similar random unannounced visits will be conducted and observations will be made.

### Statistical analysis

Descriptive analyses (mean, standard deviation, frequency, range) will be conducted for the different baseline variables in both study groups. To evaluate the success of the randomization and matching, baseline values will be analysed for differences between the intervention and control group (Chi Square, independent T-tests and/or Mann-Whitney test). Total, training and match incidence of acute injury (and its 95% CI) will be calculated by dividing the number of newly incurred acute injuries (excluding re-injuries) identified during the follow-up by the sum of volleyball exposure (total exposure, training exposure and match exposure) in hours until the first injury [14, 15]. For overuse injuries, prevalence repeatedly measured over time is considered the preferable measure [15]. Therefore, the mean prevalence (expressed as percentage) of overuse injuries repeatedly measured over time will be calculated for each 2-week period by dividing the number of participants reporting an overuse injury during that period by the total number of respondents during the same period. Then, mean prevalence (and its 95% CI) will be calculated by dividing all prevalence rates measured every 2 weeks by the number of 2-week periods. Differences in the incidence of acute injury between the intervention and control groups will be assessed using cox regression analysis (significance level set at *p* < .05; adjusted for age and/or gender). Differences in the prevalence of overuse injury between the intervention and control groups will be assessed by comparing their mean prevalence (independent T-tests and/or Mann-Whitney test). Secondary analyses will be conducted to compare the onset of acute and overuse injury between the intervention and control groups based on three levels of adherence (low, medium, high).

## Discussion

This article describes the design of a study evaluating the effectiveness of an intervention on the occurrence of finger/wrist, ankle, shoulder and knee injuries among adult recreational volleyball players. This study will be one of the first randomized prospective controlled trials in recreational volleyball that looks at the occurrence of both acute and overuse injuries.

The two major challenges of this study are related to the recruitment of a large number participants and the monitoring of both exposure and injuries. A total of 456 volleyball players across 46 teams (10 players per team on average) and two competition regions are enrolled in the study. The selection of the two largest competition regions in The Netherlands was based on practical reasons. In these two regions, a total of 2797 adult teams across 507 clubs are available, which should be sufficient to reach and enroll 456 participants. Also, holding explanatory group meetings for coaches about the purpose and procedures of the study remains more efficient when those meetings are geographically located nearby the enrolled clubs. For the monitoring of volleyball exposure and injuries, coaches and players are asked to complete an electronic questionnaire every two weeks during the whole 2017–2018 season. Even if not time consuming for most participants (likely to remain uninjured), completing a questionnaire every two weeks remains a substantial task for all participants that might play a role in the recruitment and follow-up of participants as well. However, completing such an electronic questionnaire every two weeks is needed to monitor accurately the occurrence of especially overuse injuries. In sports injury epidemiology, recording overuse injuries has always been a problem as athletes are often likely to continue their sport participation despite their complaints. In our study, the use of the reliable and valid OSTRC questionnaire will allow us to record over time both acute and overuse injuries, adding an unequivocal and unique value to the existing sports injury epidemiological evidence.

After the systematic development of ‘VolleyVeilig’, a randomised prospective controlled trial enables to assess its effectiveness on the one-season occurrence of finger/wrist, shoulder, knee and ankle injuries among recreational adult volleyball players. The results of this trial will contribute to the successful nationwide implementation of ‘VolleyVeilig’ in all volleyball clubs in The Netherlands, and ultimately to the reduction of injury occurrence.

## Abbreviations

Nevobo: Dutch Volleyball Federation
NTR: Dutch Trial Registry (Nederlands Trial Register)
OSTRC: Oslo Sports Trauma Research Centre
ZonMW: Netherlands Organization for Health Research and Development
